# Backscattering particle immunoassays in wire-guide droplet manipulations

**DOI:** 10.1186/1754-1611-2-15

**Published:** 2008-11-17

**Authors:** Jeong-Yeol Yoon, David J You

**Affiliations:** 1 Department of Agricultural and Biosystems Engineering, the University of Arizona, Tucson, AZ 85721-0038, USA

## Abstract

A simpler way for manipulating droplets on a flat surface was demonstrated, eliminating the complications in the existing methods of open-surface digital microfluidics. Programmed and motorized movements of 10 μL droplets were demonstrated using stepper motors and microcontrollers, including merging, complicated movement along the programmed path, and rapid mixing. Latex immunoagglutination assays for mouse immunoglobulin G, bovine viral diarrhea virus and *Escherichia coli *were demonstrated by merging two droplets on a superhydrophobic surface (contact angle = 155 ± 2°) and using subsequent back light scattering detection, with detection limits of 50 pg mL^-1^, 2.5 TCID_50 _mL^-1 ^and 85 CFU mL^-1^, respectively, all significantly lower than the other immunoassay demonstrations in conventional microfluidics (~1 ng mL^-1 ^for proteins, ~100 TCID_50 _mL^-1 ^for viruses and ~100 CFU mL^-1 ^for bacteria). Advantages of this system over conventional microfluidics or microwell plate assays include: (1) minimized biofouling and repeated use (>100 times) of a platform; (2) possibility of nanoliter droplet manipulation; (3) reprogrammability with a computer or a game pad interface.

## Background

**Figure 1:**
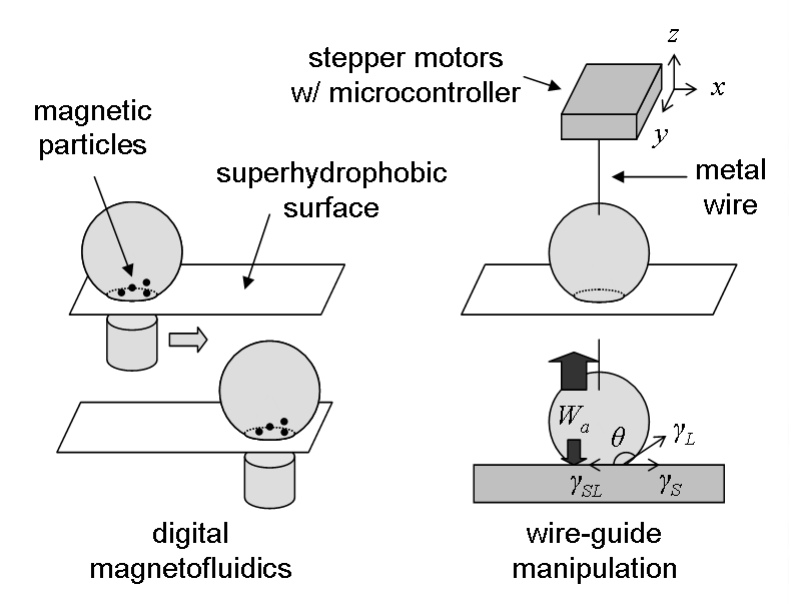
Magnetofluidics vs. wire-guide droplet manipulation.

There has been a growing interest in recent years for digital microfluidics through manipulating droplets on an open surface. Although there are some arguments regarding whether it is better to use microflows or droplets in microfluidics, it is clear that complex and reconfigurable (or reprogrammable) bioanalysis and biorecognition are only possible by using droplets [1]. There are two different types of droplet manipulations: (1) using discrete liquid plugs in pre-defined microchannels [2,3], or (2) using droplets sitting on an open, flat surface [4,5]. Although the former (liquid-plug type) has been popular in digital microfluidics, the latter (open-surface type) has more potential as its reaction protocol can be reprogrammed to whatever combination one can conceive. A couple of droplet manipulation attempts have been demonstrated on an open surface, most notably magnetofluidics (Figure 1 in the left). In magnetofluidics, droplets containing paramagnetic particles move over a superhydrophobic surface under the influence of an external magnetic field [6]. Paramagnetic particles should be designed not to interfere with biological reactions, a capability that has not been confirmed yet.

Whether we are able to manipulate a droplet or not depends primarily on the surface tension of the droplet, which is closely associated with its contact angle. Figure 1 in the bottom right graphically defines the liquid contact angle *θ*. In most cases, the liquid is water or other aqueous solution. A liquid drop sitting on a certain surface maintains its shape due to the equilibrium in its surface tensions, specifically at its three-phase borderline. At this line, three different surface tensions are in exact balance, *γ*_*SL *_(solid-liquid), *γ*_*SV *_(solid-vapor) and *γ*_*LV *_(liquid-vapor). The latter two are often abbreviated as *γ*_*S *_and *γ*_*L *_since vapor can be approximated as vacuum (hence no subscript). As shown in Figure 1, the balance of three surface tension vectors should provide the following Young equation [7]:

(1) *γ*_*SL *_= *γ*_*S *_- *γ*_*L *_cos *θ*.

The Dupré equation describes the interaction between two different materials. For a solid surface and a liquid drop, the free energy of the work of adhesion (*W*_*a*_; the adhesive energy of a liquid drop to a solid surface) is [7]:

(2) *W*_*a *_= *γ*_*S *_+ *γ*_*L *_- *γ*_*SL*_.

Combining the Young and Dupré equations yields the following definition on the work of adhesion (Young-Dupré equation):

(3) *W*_*a *_= *γ*_*L *_(1 + cos *θ*).

Equation (3) tells us how much work should be provided to overcome *W*_*a *_of a droplet to a surface, which is primarily a function of *θ *(*γ*_*L *_is constant if the liquid is mostly water).

In magnetofluidics, it is difficult to calculate the exact magnetic energy between paramagnetic particles and a magnetic bar shown in Figure 1 in the left. Since magnetofluidics has not been successful with conventional plastic surfaces, we can assume such magnetic energy is generally too weak to overcome *W*_*a *_for many conventional surfaces. Therefore, a superhydrophobic surface (whose water contact angle *θ *is 150° or higher) is needed in order to minimize both the contact area and *W*_*a *_for a droplet (the latter is often defined as "frictional force").

In both cases, external electric or magnetic fields may affect the behavior of biomolecules, thus affecting subsequent bioanalysis and biorecognition. In fact, optical detection was never demonstrated for magnetofluidics. A simpler way for manipulating droplets on a flat surface is needed to minimize the complications described above. A clean, metal wire (water contact angle *θ *< 10°) may be inserted into a droplet to guide its movement on a surface. However, the *W*_*a *_of a droplet to a metal wire is simply too small to overcome the *W*_*a *_to a flat surface due to the small contact area between the droplet and wire. Since *W*_*a *_= *γ*_*L *_(1 + cos *θ*), where *γ*_*L *_is the liquid surface tension, a very large *θ *may make this movement possible. In this work, we used a superhydrophobic surface with *θ *= 155° for droplet manipulations. Linear movements and subsequent merging of two droplets were attempted.

We demonstrated this droplet merging for particle immunoassays (more specifically, latex immunoagglutination assays). One droplet contained antibody-conjugated latex particles and the other contained target antigens. Three different target antigens were tested: mouse immunoglobulin G (mIgG; model protein), bovine viral diarrhea virus (BVDV; model virus) and *Escherichia coli *(*E. coli*; model bacterium). Antibody-antigen binding caused the latex particles to agglutinate, leading to the increased extent of light scattering, which was used for detection [8].

Light scattering detection is the most appropriate sensing modality for latex immunoagglutination, as there is no fluorescent dye in the system. Incident beam of light is scattered to all directions by latex particles, which is usually elastic (i.e. the wavelength of incident light is the same as that of scattered light). Incident and scattered light can be distinguished by locating a light detector not in parallel with the light source but at a certain angle (15°, 30°, 45°, 90° and 180° are commonly used; we used 180° which is back scattering). We used microparticles (920 nm in diameter) whose light scattering roughly follows the Mie theory [9]. In this regime, light scattering intensity is a strong function of the particle size rather than the particle number; hence latex immunoagglutination leads to larger extent of light scattering [10]. We maintained the intensity of a light source as constant, i.e. static light scattering.

## Results and discussion

### Wire-guide manipulations for open-surface digitalmicrofluidics

**Figure 2:**
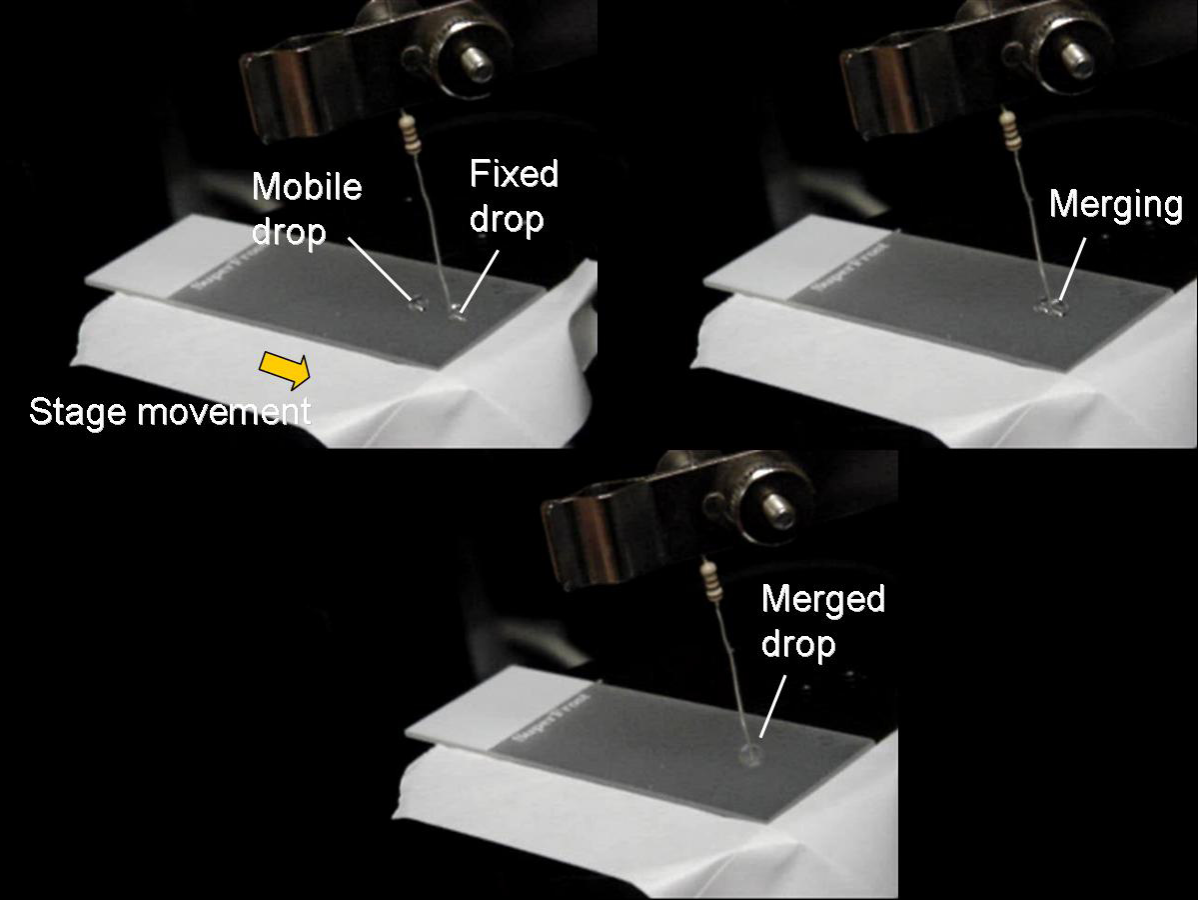
**Linear movements and merging of two droplets**. On a superhydrophobic surface; A resistor was used as a metal wire but no voltage was applied.

Figure 2 shows the merging of two 10 μL droplets of deionized water (from Millipore Simplicity, Molsheim, France) on a superhydrophobic surface, clearly demonstrating two basic droplet manipulations: moving and merging. Movements were repeatable over the same line more than 10 times, regardless of the content of droplets. Movements were also successful for 5 and 20 μL droplets. Droplets were removed simply by tilting the surface and no further cleaning/rinsing was performed. Similar experiments were performed using polystyrene surfaces (plastic Petri dishes from Fisher Scientific; Pittsburg, PA, USA), but movements were not successful.

The water contact angles (*θ*) were 155 ± 2° on superhydrophobic surfaces, and those for 0.02% w/v antibody-conjugated particles and target solutions were not significantly different from those of water (154° to 156° with standard deviations of 2°), as measured using FTÅ200 (from First Ten Ångstroms, Portsmouth, VA, USA). The liquid surface tensions (*γ*_*L*_) were also similar regardless of solution, and were measured at 73 ± 1 mN/m also using FTÅ200. The contact area of 10 μL droplet was measured as 2.0 ± 0.2 mm^2 ^(by FTÅ 200). Therefore, the work of adhesion (*W*_*a*_) of 10 μL droplets to the superhydrophobic surfaces is:

(73 mN/m) (1 + cos 155°) (2.0 mm^2^) = 14 nJ.

Similar analysis can be made for the metal wires. (Note that a resistor was used merely as a metal wire; no voltage was applied.) Since the diameter of the metal wire was 0.5 mm and the insertion depth was 2 mm, the contact area between the metal wire and the droplet was 0.39 mm^2^. Since most clean metal surfaces have a water contact angle of 10° [11], *W*_*a *_can be estimated as:

(73 mN/m) (1 + cos 10°) (0.39 mm^2^) = 57 nJ,

which is larger than that of a droplet to a superhydrophobic surface, consequently enabling droplet movement.

The water contact angles on the polystyrene surfaces were 91 ± 5° and the contact area of 10 μL droplets were 10.5 ± 0.4 mm^2 ^(again using FTÅ200). The *W*_*a *_of 10 μL droplet to a polystyrene surfaces is:

(73 mN/m) (1 + cos 91°) (10.5 mm^2^) = 750 nJ,

indicating a droplet cannot be moved with a metal wire on a polystyrene surface.

### Particle immunoassays in open-surface digital microfluidics

**Figure 3:**
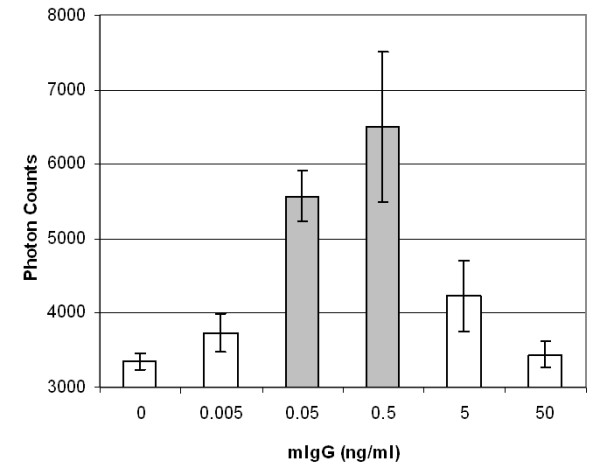
**Backscattering of latex immunoagglutination for mIgG**. Average of three experiments. Error bars are standard deviations. Grey-colored bars indicate significant differences (p < 0.05) from a blank.

**Figure 4:**
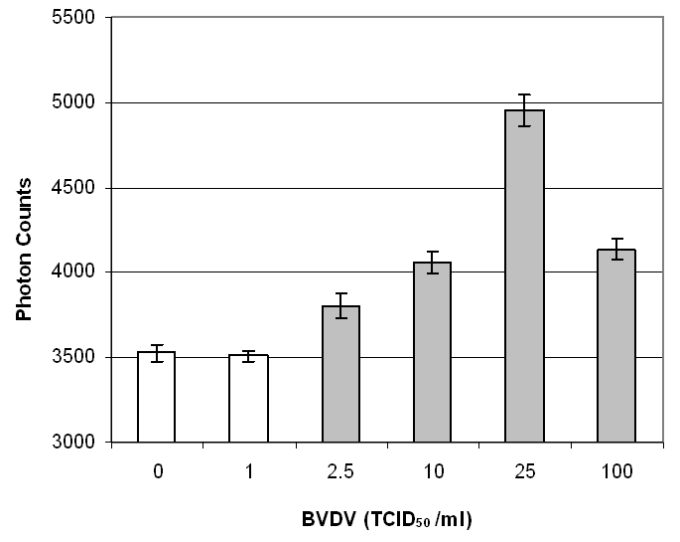
**Backscattering of latex immunoagglutination for BVDV**. Average of three experiments. Error bars are standard deviations. Grey-colored bars indicate significant differences (p < 0.05) from a blank.

**Figure 5:**
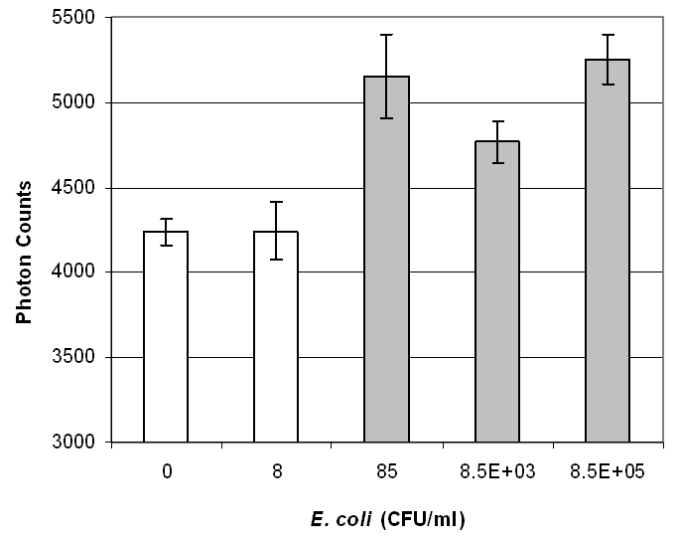
**Backscattering of latex immunoagglutination for *E. coli***. Average of three experiments. Error bars are standard deviations. Grey-colored bars indicate significant differences (p < 0.05) from a blank.

Figures 3, 4 and 5 show the maximum light intensities taken from the merged droplets. All results are the averages of three different experiments (i.e. each taken from different merged droplets). Error bars indicate standard deviations. Paired, two-tailed *t*-tests were performed by comparing each dilution with a blank (10 mM PBS). Dilutions with significant differences from the blank are indicated by the grey color. The detection limit for mouse immunoglobulin G (mIgG) was 50 pg mL^-1^, equivalent to 0.5 pg of mIgG in a 10 μL target droplet.

The detection limit for bovine viral diarrhea virus (BVDV) was 2.5 TCID_50 _mL^-1 ^(TCID_50 _= 50% tissue culture infectious dose), equivalent to 0.025 TCID_50 _for a 10 μL target drop. Although this detection limit is much lower than many other assays, it is still a possible number since there are at least hundreds or up to a few tens of thousands of virus particles in 1 TCID_50_. The detection limit for *E. coli *was 85 CFU mL^-1 ^(CFU = colony forming unit), equivalent to 0.85 CFU for a 10 μL target drop and subsequently less than one bacterium (assuming 1 CFU = 1 viable cells). We have to recall that BVDV solutions were washed by centrifuging, eliminating a bulk of cell fragments and free antigens that may be recognized by anti-BVDV (specific or non-specific). We may attribute the smaller standard deviations for BVDV compared to mIgG and *E. coli *to this washing treatment. In contrast, *E. coli *solutions were not washed by centrifuging, and they possibly contained many cell fragments and free antigens [12]. This indicates that the number of *E. coli *antigens may be much larger than one when the viable cell count = 1.

All three figures follow the so-called Heidelberger-Kendall curve [10], characterized by initial increase at lower target (antigen) concentrations, followed by a decrease at higher concentrations. The left-hand side (initial increase) can serve as a calibration curve, as well as providing a linear range of immunoassay. The right-hand side (decrease at higher concentrations) represents the antigen-excess region (i.e. too much target antigens for a fixed amount of antibodies bound to the particles), which inhibits antibody-antigen binding. The apparent advantage of this droplet manipulation is that the liquids are in minimal contact with solid surfaces. The bottom superhydrophobic surface consists largely of air pockets and the contact area of a top wire is extremely small. This minimized contact reduces biomolecular adsorption (biofouling), enabling repeated use of a platform. In fact, a single superhydrophobic surface could be repeatedly used for > 100 times for all droplets used in this experiments (blank and target solutions as well as antibody-conjugated particle suspensions). In this sense, we believe this setup can replace the standard microwell plate assays, where liquids are in direct contact with surfaces (thus resulting in more biofouling). Liquid volume is smaller than that of microwell plate assays, which may be further lowered to nanoliter scale (presumably in oil immersion to prevent rapid evaporation).

### Motorizing and programming the wire manipulations

**Figure 6:**
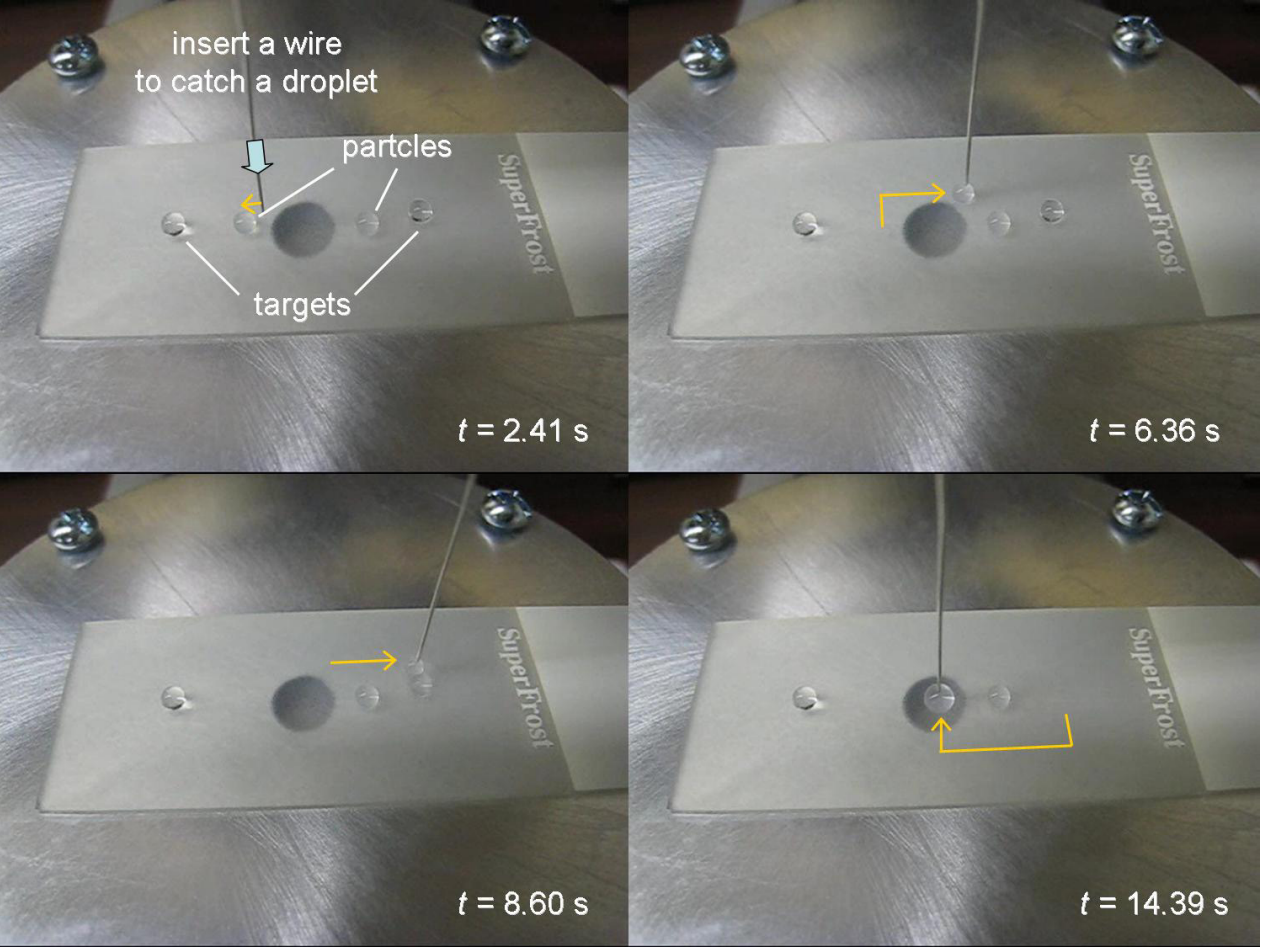
**Snapshots from pre-programmed droplet movements**. Top left: taking a 10 μL droplet of 0.02% (w/v) antibody-conjugated particle suspension by inserting a wire into the droplet; Top right: its linear movements; Bottom left: merging with a 10 μL target droplet; Bottom right: linear movements of this merged 20 μL droplet towards the detection site. Complete movie is available as Additional file 1.

To demonstrate the automation possibility of these wire-guide droplet manipulations, we constructed a three-axis droplet manipulator using stepping motors and subsequent microcontrollers. Figure 6 shows the snapshots of pre-programmed movements of a droplet, starting from (1) taking a 10 μL droplet of 0.02% (w/v) antibody-conjugated particle suspension by inserting the wire into the droplet, (2) its linear movements, (3) merging with a 10 μL target droplet, and (4) linear movements of this merged 20 μL droplet towards the detection site. Complete movie is also available as Additional file 1, which also shows the movements controlled by a game pad.

**Figure 7:**
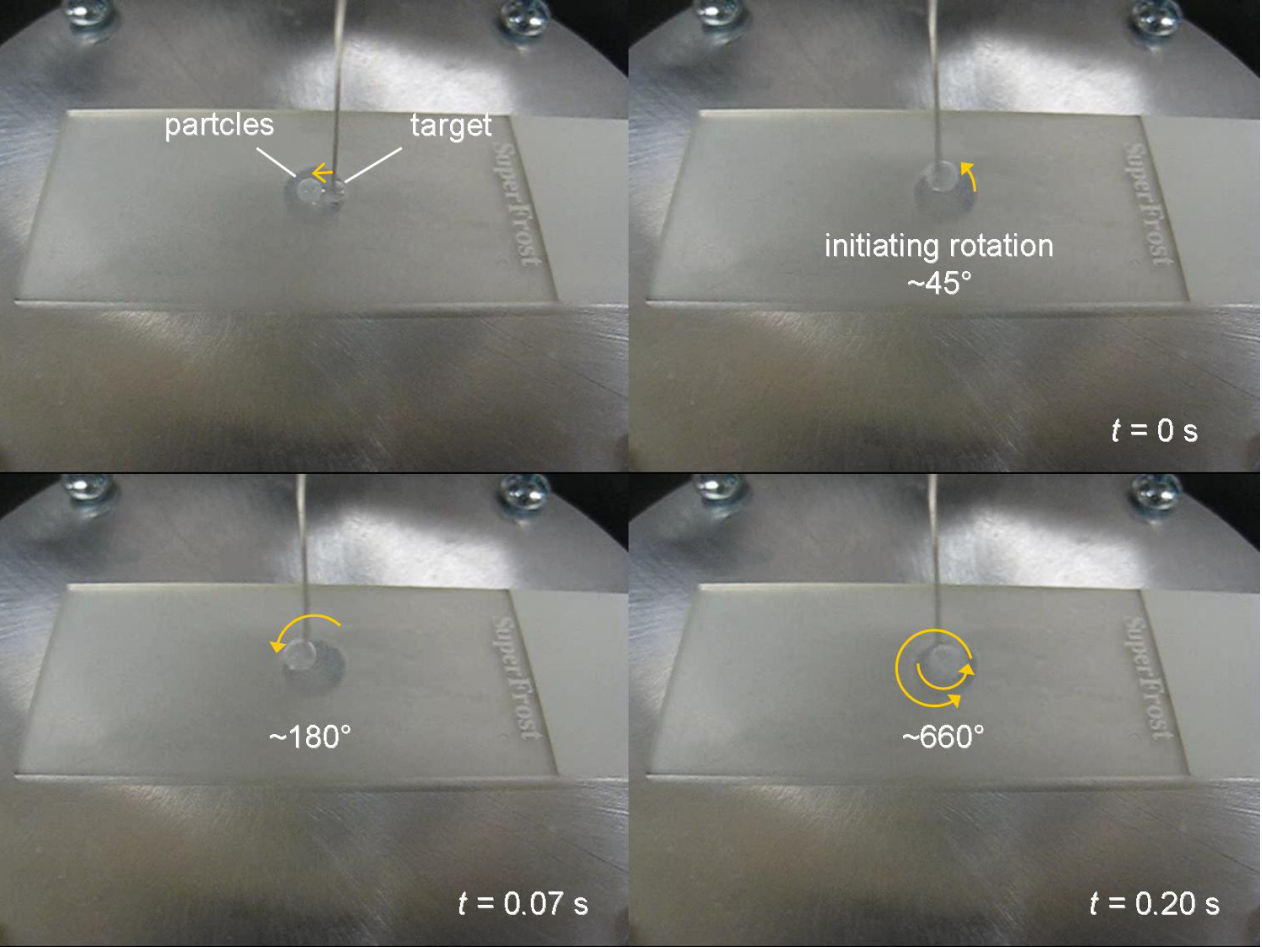
**Snapshots from rapid mixing of a merged droplet**. Complete movie is available as Additional file 1.

We also demonstrated the rapid mixing of a merged droplet, by mechanically vibrating the wire with a vibration motor. Figure 7 shows the snapshots of this movie. Complete movie is also available as Additional file 1. The total cost of materials and supplies were less than $230 (excluding the experimenter's labor), which demonstrates the feasibility of this new droplet manipulation. Additionally, smaller stepper motors and integrated circuits (all commercially available) could greatly reduce the overall size of this setup.

These automated and programmed droplet manipulations eventually lead to "reprogrammable" digital microfluidics, where the reaction protocols can be altered by the user's program input or simply by the user's control of a game pad. Potential applications include, but are not limited to: potentiometric/conductometric titration, immunoassay, serial dilution, polymerase chain reaction (PCR) and single cell analysis.

## Conclusion

To summarize, a proof-of-concept was demonstrated for the wire-guide droplet manipulations and subsequent bioanalysis. Detection limits were extremely low compared to the other immunoassay demonstrations in conventional microfluidics; > 1 ng mL^-1 ^for proteins [13-15], > 100 TCID_50 _mL^-1 ^for viruses [16,17], > 100 CFU mL^-1 ^for bacteria [18,19]. We hope this new method would substitute magnetofluidics by minimizing or even eliminating their complications, such as biomolecular adsorption, possible interferences by external electrical or magnetic field. The simplicity of this wire-guide manipulation is also demonstrated with automated, programmed movements of droplets.

## Methods

### Target solutions

Immunoglobulin G from murine serum, or mouse immunoglobulin G (mIgG), was chosen as a model protein (from Sigma-Aldrich; St. Louis, MO, USA). It was dissolved in 10 mM, pH 7.4 phosphate buffered saline (PBS) and various dilutions were made from a single stock solution. Bovine viral diarrhea virus (BVDV) was selected as a model virus pathogen. As its name indicates, BVDV causes diarrhea in cattle, leading to productivity loss and death. BVDV was cultured in Madin-Darby bovine kidney cells (MDBK) with appropriate tissue culture media (containing 5–10% fetal calf serum), followed by cell denaturation and centrifugal washing (from NVRQS; National Veterinary Research and Quarantine Service; Anyang, South Korea). Various dilutions were made from this stock solution; the tissue culture infectious dose 50 (TCID_50_) value was provided by the manufacturer (NVRQS). Finally, *E. coli *was selected as a model bacterial pathogen. Lyophilized *E. coli *K-12 powder was purchased from Sigma-Aldrich and cultured in brain heart infusion broth (from Remel; Lenexa, KS, USA) at 37°C for 20 h. The colony forming units (CFU) was evaluated by plating some of the above dilutions on eosin methylene blue agar (from DIFCO; Lawrence, KS, USA), incubating at 37°C for 20 h and counting the number of colonies with a light microscope (from Nikon; Tokyo, Japan). Serial dilutions from the stock solution were made using 10 mM phosphate buffered saline (PBS).

### Antibody-conjugated particles

Latex particles were purchased from Bangs Laboratories (Fishers, IN, USA). These particles were highly carboxylated, with 10.3 Å^2 ^parking area per carboxyl surface group, and a mean diameter of 920 nm according to the manufacturer's specifications. These particles should be mixed faster with target solution through their higher diffusivity, without using any surfactants, as we have recently demonstrated in microfluidic platforms [20]. Maximum signals were obtained at 2 min, and all data points were taken at 2 min after merging two droplets. No significant evaporation was observed for this time frame. The particles were conjugated with three different polyclonal antibodies: anti-mIgG from Sigma-Aldrich, anti-BVDV from Jeno Biotech (Chuncheon, South Korea) and anti-*E. coli *from Abcam (Cambridge, MA, USA). These antibodies were conjugated to the particles by physical adsorption following the same protocol published previously [12]. Antibody-conjugated particles were centrifuged twice, until no antibodies could be found in supernatants with the absorbance measurements at 280 nm. The surface coverage of antibodies was set to approximately 33% of its maximum possible value [21]. The solid content of antibody-conjugated particles was 0.02% w/v.

### Droplet manipulations

Superhydrophobic surfaces made from nanocoatings of fluoropolymer on standard glass microscope slides were purchased from Surface Innovations (Durham, England). These nanocoatings create air pockets and put a microdrop in metastable Fakir state [22].

**Figure 8:**
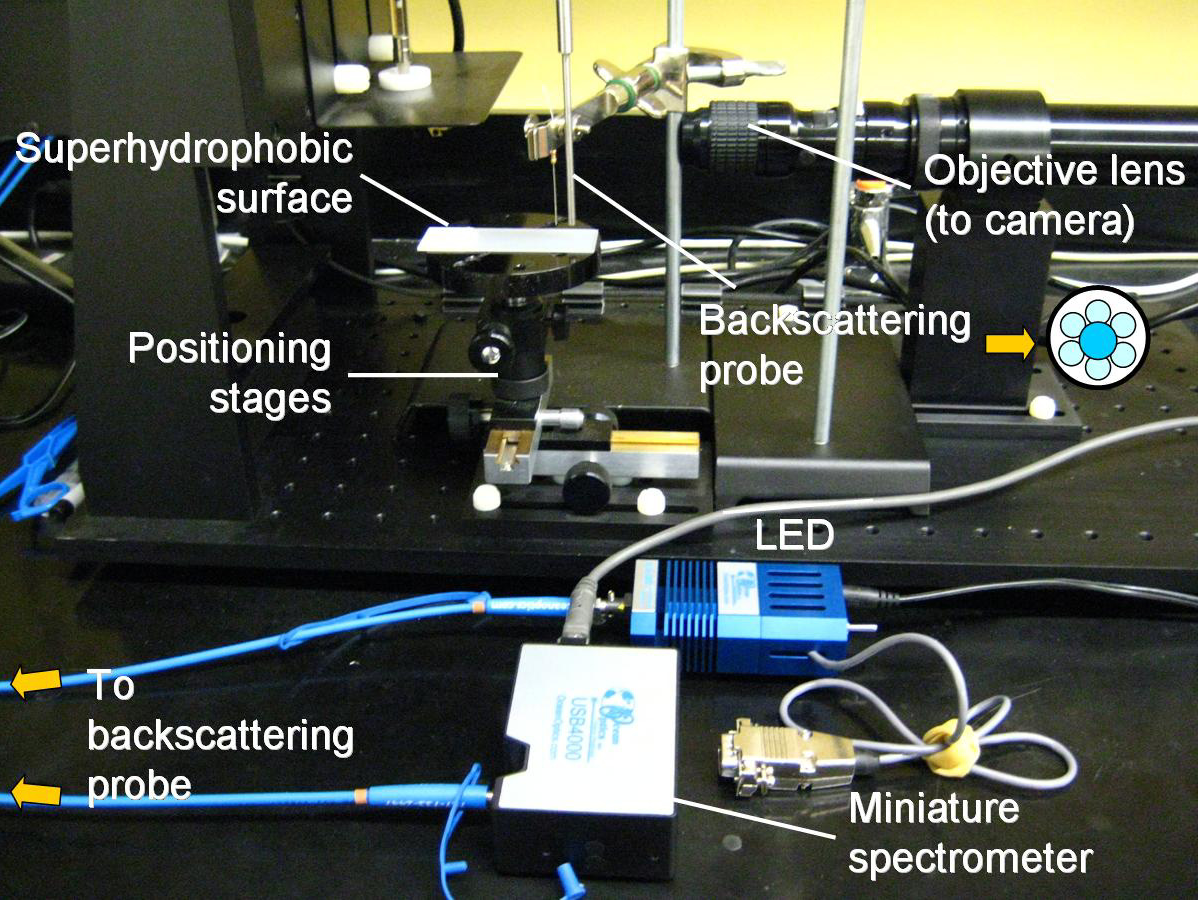
Experimental setup for manual manipulation and backscattering detection.

Figure 8 shows the experimental setup for the droplet manipulations and subsequent backscattering detection. Briefly, 10 μL each particle and target solution droplets were placed on a single superhydrophobic surface that was sitting on the positioning stages (from Edmund Optics; Blackwood, NJ, USA) capable of X-, Y- and Z-movements. A metal wire was inserted into the rightmost particle droplet by adjusting the positioning stages. The positioning stage was moved to the right so that the target droplet moved to the right with the superhydrophobic surface, while the particle droplet was fixed with a metal wire. Once the two droplets were merged, the positioning stage was moved downward to remove the metal wire. The merged droplet was then moved to the right so that it was positioned underneath the backscattering probe. The backscattering probe with optical fibers was purchased from Ocean Optics (Dunedin, FL, USA); it consists of a core-shell bundle of optical fibers and is shown on the right in Figure 8. A single fiber at the core delivers 375-nm light from a light emitting diode (LED; LS-450 from Ocean Optics) and six fibers at the shell side collect 180° backscattered light and transfer the signal to a USB4000 miniature spectrometer (from Ocean Optics). The positioning stages were moved along the X-, Y- and Z-directions to collect the maximum light intensity. SpectraSuite software (from Ocean Optics) was used to collect light intensities at 375 nm, without averaging over adjacent wavelengths (i.e. no smoothing). The integration time for data collection was 100 ms.

### Motorizing and programming the droplet manipulations

**Figure 9:**
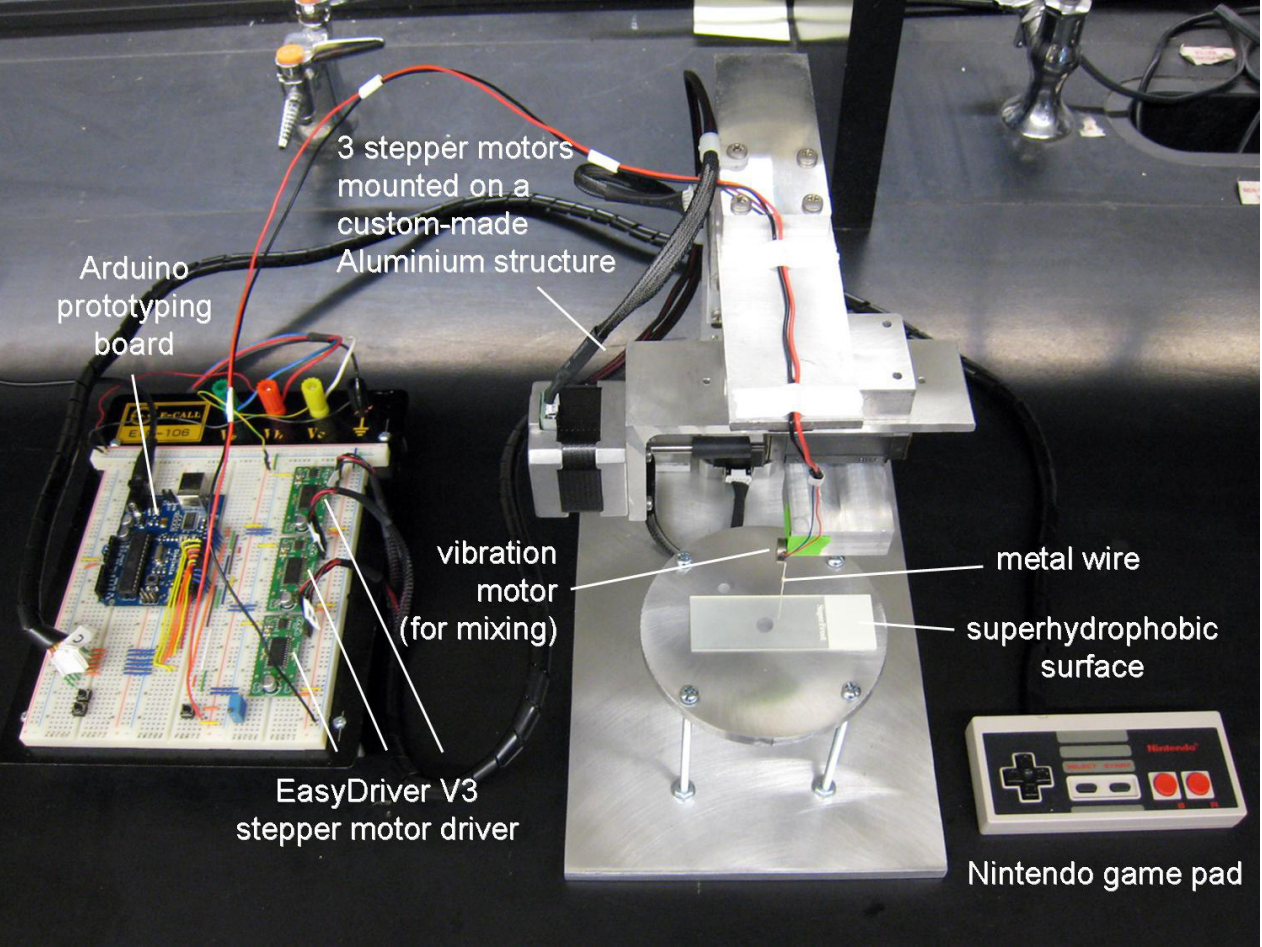
Experimental setup for automated manipulation.

Figure 9 shows the experimental setup for motorized and programmed manipulations of droplets. The computer controlled three-axis droplet manipulator utilizes a microcontroller with a USB interface, 1.8° stepper motors, and an aluminium structure with THK SSR25 linear bearings. The aluminium components were machined from 6061 alloy blocks and extruded t-channels via three-axis Lagun vertical mill to within ten-thousandths of an inch accuracy. The t-channels serve as a bracket to create a rigid body with the stepper motors and linear bearings. Spacers attached between the linear bearings allow the output shaft of the stepper motors to rotate a threaded bolt that passes through it, transforming rotational motion to linear displacement along the direction of the output shaft. The microcontroller is an Arduino, an open-source electronics prototyping board manufactured by SmartProjects (Italy). The stepper motors are two-phase with a 1.8° step angle by SparkFun Electronics (Boulder, CO, USA). This means the stepper motors can complete 200 steps in a full 360° rotation. When combined with the EasyDriver V3 Stepper Motor Driver, also by SparkFun Electronics, the A3967 integrated circuit allows for microstepping, which allows the stepper motors to complete 8 microsteps per step. Thus, the stepper motors are capable of undergoing 1600 individual steps per rotation. The thread pitch is 0.79375 mm, therefore with 1600 steps per rotation, the theoretical displacement per step is 0.496 μm. With 200 steps per rotation, the displacement per step is 3.96 μm.

The Arduino board outputs simultaneously to three stepper motor driver boards, one per axis, through digital outputs integrated on the board. An original Nintendo controller wired into the digital inputs on the Arduino board allows the user to control the wire's position on-the-fly. The directional pad controls the X and Y axis movements, and the A and B buttons control the Z axis movement (i.e. insertion/retraction of a wire into a droplet). In conjunction with the controller, the system can be easily programmed to complete certain movements precisely and with consistency each time. The stage can be adjusted to ensure level surface conditions when working with superhydrophobic surfaces.

## Competing interests

The authors declare that they have no competing interests.

## Authors' contributions

JYY conceived the original idea and performed the experiments of manual droplet manipulations, particle immunoassays and subsequent data analyses. DJY designed and performed the experiments of automated droplet manipulations.

## Supplementary Material

Additional file 1 **Real-time movie for (1) pre-programmed movements, (2) game-pad manipulations, and (3) rapid mixing.** The images of Figures 6 and 7 were taken from the first and third sections of this movie. Click here for file
